# Supplementary material for: Sorting at embryonic boundaries requires high heterotypic interfacial tension
Source: Nat Commun. 2017 Jul 31;8:157. doi: 10.1038/s41467-017-00146-x (PMC5537356; doi:10.1038/s41467-017-00146-x)
Supplement: Supplementary file 2 — Supplementary Software 1 [file 41467_2017_146_MOESM2_ESM.zip › PottsModel/SrcPottsModel/doc/model/Coordinates.html]

Coordinates


JavaScript is disabled on your browser.


Skip navigation links


- Overview
- Package
- Class
- Use
- Tree
- Deprecated
- Index
- Help

- Prev Class
- Next Class

- Frames
- No Frames

- All Classes

- Summary:
- Nested |
- Field |
- Constr |
- Method

- Detail:
- Field |
- Constr |
- Method


model

## Interface Coordinates

- All Known Implementing Classes:
  :   CartesianCoordinates, Hexagon.AxialCoordinates, Hexagon.CubeCoordinates, Hexagon.OffsetCoordinates

  ---

    

  ```
  public interface Coordinates
  ```

- - ### Method Summary

    All Methods Instance Methods Abstract Methods

    | Modifier and Type | Method and Description |
    | --- | --- |
    | `double` | `getX()` |
    | `double` | `getY()` |
    | `double` | `getZ()` |
    | `void` | `setX(double pX)` |
    | `void` | `setY(double pY)` |
    | `void` | `setZ(double pZ)` |
    | `java.lang.String` | `toString()` |

- - ### Method Detail


    - #### getX

      ```
      double getX()
      ```


    - #### getY

      ```
      double getY()
      ```


    - #### getZ

      ```
      double getZ()
      ```


    - #### toString

      ```
      java.lang.String toString()
      ```

      Overrides:
      :   `toString` in class `java.lang.Object`


    - #### setX

      ```
      void setX(double pX)
      ```


    - #### setY

      ```
      void setY(double pY)
      ```


    - #### setZ

      ```
      void setZ(double pZ)
      ```


Skip navigation links


- Overview
- Package
- Class
- Use
- Tree
- Deprecated
- Index
- Help

- Prev Class
- Next Class

- Frames
- No Frames

- All Classes

- Summary:
- Nested |
- Field |
- Constr |
- Method

- Detail:
- Field |
- Constr |
- Method
